# The Physical Spectrum of a Driven Jaynes–Cummings Model

**DOI:** 10.3390/e28010127

**Published:** 2026-01-21

**Authors:** Luis Medina-Dozal, Alejandro R. Urzúa, Irán Ramos-Prieto, Ricardo Román-Ancheyta, Francisco Soto-Eguibar, Héctor M. Moya-Cessa, José Récamier

**Affiliations:** 1 Instituto de Ciencias Físicas, Universidad Nacional Autónoma de México, Avenida Universidad s/n, Col. Chamilpa, Cuernavaca 62210, Morelos, Mexico; luis.medina@icf.unam.mx (L.M.-D.); alejandro@icf.unam.mx (A.R.U.); pepe@icf.unam.mx (J.R.); 2 Instituto Nacional de Astrofísica, Óptica y Electrónica, Calle Luis Enrique Erro No. 1, Santa María Tonantzintla, Puebla 72840, Mexico; iran@inaoep.mx (I.R.-P.); feguibar@inaoep.mx (F.S.-E.); 3 Centro de Física Aplicada y Tecnología Avanzada, Universidad Nacional Autónoma de México, Boulevard Juriquilla 3001, Querétaro 76230, Mexico

**Keywords:** driven Jaynes–Cummings model, physical spectrum, invariant approach, two-time correlation functions, Eberly–Wodkiewicz spectrum

## Abstract

We analyze the time-dependent physical spectrum of a driven Jaynes–Cummings model in which both the two-level system and the quantized cavity mode are subject to coherent classical driving. The time-dependent Hamiltonian is mapped, via well-defined unitary transformations, onto an effective stationary Jaynes–Cummings form. Within this framework, we derive closed-form expressions for the two-time correlation functions of both the atomic and field operators. These correlation functions are subsequently used to evaluate the time-dependent physical spectrum according to the Eberly–Wódkiewicz definition, which properly accounts for finite spectral resolution and transient emission dynamics. We show that the external driving leads to substantial modifications of the atomic spectral response, including controllable frequency shifts and asymmetric line shapes. Importantly, we identify a regime in which the driving parameters are chosen such that the coherent displacement induced in the cavity field exactly cancels out the initial coherent amplitude. In this limit, the system dynamics reduce to that of an effectively vacuum-initialized Jaynes–Cummings model, and the standard vacuum Rabi splitting is recovered. This behavior provides a clear and physically transparent interpretation of the spectral features as arising from coherent field displacement rather than from modifications of the underlying atom–cavity coupling.

## 1. Introduction

The Jaynes–Cummings model (JCM) is a cornerstone of quantum optics, providing a fundamental and mathematically rigorous description of light–matter interactions at the quantum level [1,2]. Since its conceptual formulation, the model has been experimentally validated on various physical platforms, most notably in the strong-coupling regime of circuit quantum electrodynamics [3,4]. By modeling the interaction between a two-level system and a single quantized electromagnetic mode, the JCM has served as an ideal paradigm for investigating non-classical phenomena, including the periodic collapse and revival of atomic inversion [5], vacuum Rabi splitting [6,7,8,9], and the generation of Schrödinger cat states [10]. Over the decades, the framework has been extended to accommodate dissipation and decoherence in lossy cavities [10,11], as well as nonlinear interactions, such as intensity-dependent and multiphoton generalizations of the JCM [12], experimental realizations of two-photon processes in circuit QED [13], and the engineering of nonlinear coherent and nonclassical field states [14]. A comprehensive overview of these nonlinear extensions and their modern research directions can be found in [15]. Modern developments have further expanded the reach of the JCM, scaling it to many-body systems via circuit quantum electrodynamics lattices for quantum simulation [16]. This expansion has also explored generalized frameworks, such as the anisotropic quantum Rabi model, which allows the simultaneous treatment of the Jaynes–Cummings and anti-Jaynes–Cummings regimes [17]. Recent spectroscopic studies of nonlinear variants of the JCM have also revealed novel coupling regimes and spectral asymmetries [18]. A particularly versatile generalization is the driven JCM, where classical external fields act on the atom, cavity mode, or both, enabling control over the quantum state of the system [19,20]. Recently, exact analytical solutions for the time-dependent JCM under external driving have been reported [21], along with studies addressing the effects of dissipation on squeezed light and entanglement [22], as well as the robustness of geometric phases in the presence of dissipation [23]. These contributions extend the understanding of the dynamical properties of open and nonstationary quantum systems.

Solving the time-dependent Schrödinger equation for systems characterized by non-stationary interactions remains a difficult task, often requiring perturbative analytical expansions or specialized numerical techniques. However, for the JCM driven with simultaneous atomic and field classical driving, an exact analytical solution has recently been established through a dynamical invariant approach [24]. Based on the theory of time-dependent invariants [25], this framework uses a sequence of unitary transformations to map the complex time-dependent Hamiltonian to a solvable stationary form [24]. Within this formalism, the specific unitary transformations not only determine the dynamical invariant but also provide a mechanism to either factorize the Hamiltonian’s temporal dependence or eliminate it in a suitable rotating frame, thereby granting non-perturbative access to the system’s complete state vector [26,27].

On the other hand, while the state vector contains all dynamical information, extracting experimentally accessible observables requires careful consideration of the measurement process, particularly for non-stationary signals where phase-dependent fluctuations play a crucial role [28]. Conventional quantities like the atomic inversion and mean photon number provide partial insights into the energy exchange dynamics and often mask the fine spectral details of transient phenomena. To fully characterize the time-frequency properties of the emission, one must adopt the physical spectrum of light as defined by Eberly and Wódkiewicz [29], which explicitly accounts for the finite bandwidth and response time of a photodetector [30,31]. This operational approach has been crucial in elucidating several spectral signatures in quantum optics [6,7,8,9,32] and quantum thermodynamics [33,34]. Moreover, studies of unconventional photon-blockade mechanisms and non-Markovian effects have shown that nonreciprocal parametric drives and memory-induced dynamics can substantially modify photon statistics and blockade phenomena [35,36]; quantitative measures of non-Markovianity provide useful diagnostics to characterize these memory effects and their impact on spectral and statistical observables [37].

In this work, we investigate the time-dependent physical spectrum of a fully driven JCM. We derive exact analytical expressions for the two-time correlation functions of both the atomic and field subsystems, enabling a precise mapping of the spectral signal as it evolves in time. Our results show that the external driving induces a coherent displacement of the cavity field [24], which acts to fundamentally reshape the spectrum, a phenomenon that has also been explored recently in the context of the nonlinear JCM [18]. This dynamic restructuring manifests itself as tunable asymmetries and shifts in the spectral peaks, offering a controllable spectral signature of the driven light–matter interaction.

Our paper is organized as follows. Section 2 outlines the theoretical model and reviews the invariant method used to diagonalize the driven Hamiltonian. In Section 3, we derive the exact expressions for the time-dependent physical spectrum of the atom and the field, highlighting the spectral signatures of the external drive. Section 4 provides concluding remarks.

## 2. The Driven Jaynes–Cummings Model

**Figure 1:**
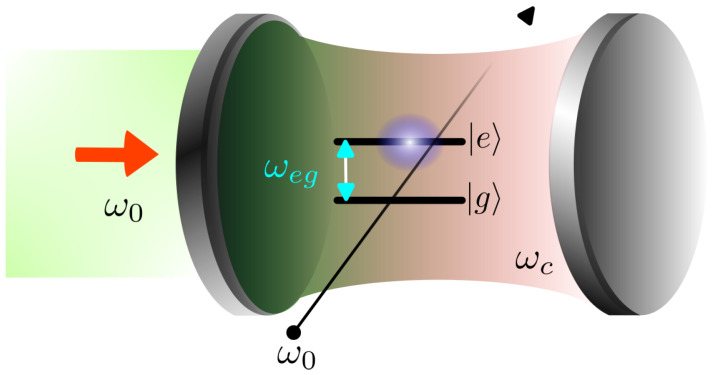
Schematic representation of a driven Jaynes–Cummings model. A two-level atom with transition frequency $\omega_{eg}$ is coupled to a single-mode cavity field of frequency $\omega_{c}$. Both the atom and the cavity mode are simultaneously driven by an external classical field of frequency $\omega_{0}$.

Consider a system composed of a two-level atom, with a ground state |g〉, an excited state |e〉, and a transition frequency $\omega_{eg}$, interacting with a single quantized electromagnetic field mode of frequency $\omega_{c}$. The system is enclosed within a high-quality cavity and is simultaneously driven by an external classical frequency field $\omega_{0}$ that couples to both atomic and photonic degrees of freedom, as schematically illustrated in Figure 1. Under the dipole and rotating-wave approximations, the time-dependent Hamiltonian in the laboratory frame is given by [19,24] (ℏ=1), written as follows:

(1) $$\begin{array}{cc} {\hat{H}}_{\mathrm{DJCM}}\left(t\right) & =\omega_{c}\;{\hat{a}}^{†}\hat{a}+\frac{\omega_{eg}}{2}{\hat{\sigma }}_{z}+g\left({\hat{\sigma }}_{+}\hat{a}+{\hat{\sigma }}_{-}{\hat{a}}^{†}\right)\\ \; & \;\;\;+\zeta \left({\hat{\sigma }}_{-}e^{i\omega_{0}t}+{\hat{\sigma }}_{+}e^{-i\omega_{0}t}\right)+\xi \left(\hat{a}e^{i\omega_{0}t}+{\hat{a}}^{†}e^{-i\omega_{0}t}\right), \end{array}$$

where *g* denotes the strength of the atom-cavity coupling, while ζ and ξ represent the driving amplitudes for the atom and the field, respectively. The field annihilation and creation operators, $\hat{a}$ and ${\hat{a}}^{†}$, satisfy the commutation relation $[\hat{a},{\hat{a}}^{†}]=1$. The atomic components are described by the pseudo-spin operators ${\hat{\sigma }}_{z}=|e\rangle \langle e|-|g\rangle \langle g|$, ${\hat{\sigma }}_{+}=|e\rangle \langle g|$, and ${\hat{\sigma }}_{-}=|g\rangle \langle e|$, which obey the standard commutation relations $\left[{\hat{\sigma }}_{+},{\hat{\sigma }}_{-}\right]={\hat{\sigma }}_{z}$ and $\left[{\hat{\sigma }}_{z},{\hat{\sigma }}_{\pm }\right]=\pm 2{\hat{\sigma }}_{\pm }$.

Following the invariant-based approach detailed in [24], we first make a transition to a rotating frame via the time-dependent unitary transformation $\hat{T}\left(t\right)=\exp\left[i\omega_{0}t\left({\hat{a}}^{†}\hat{a}+{\hat{\sigma }}_{z}/2\right)\right]$. This procedure eliminates the explicit time dependence of the driving terms, yielding the Hamiltonian, written as follows:

(2) $$\begin{array}{cc} {\hat{H}}_{T} & =\hat{T}\left(t\right){\hat{H}}_{\mathrm{DJCM}}\left(t\right){\hat{T}}^{†}\left(t\right)-i\left(\frac{\partial \hat{T}\left(t\right)}{\partial t}\right){\hat{T}}^{†}\left(t\right)\\ \; & =\Delta_{c}\;{\hat{a}}^{†}\hat{a}+\frac{\Delta_{eg}}{2}{\hat{\sigma }}_{z}+g\left({\hat{\sigma }}_{+}\hat{a}+{\hat{\sigma }}_{-}{\hat{a}}^{†}\right)+\zeta \left({\hat{\sigma }}_{-}+{\hat{\sigma }}_{+}\right)+\xi \left(\hat{a}+{\hat{a}}^{†}\right), \end{array}$$

where $\Delta_{c}=\omega_{c}-\omega_{0}$ and $\Delta_{eg}=\omega_{eg}-\omega_{0}$ are the cavity and atomic detunings, respectively. To simplify the above Hamiltonian, the linear driving terms can be eliminated by applying the displacement operator $\hat{D}\left(\alpha \right)=\exp\;\left[\alpha \left({\hat{a}}^{†}-\hat{a}\right)\right]$ [38], with α∈R, which acts on the field operators as $\hat{D}\left(\alpha \right)\hat{a}{\hat{D}}^{†}\left(\alpha \right)=\hat{a}-\alpha$ and $\hat{D}\left(\alpha \right){\hat{a}}^{†}{\hat{D}}^{†}\left(\alpha \right)={\hat{a}}^{†}-\alpha$. This transformation produces constant and linear contributions in the new displaced Hamiltonian, which reads as follows:

(3) $$\begin{array}{cc} {\hat{H}}_{D} & =\hat{D}\left(\alpha \right){\hat{H}}_{T}{\hat{D}}^{†}\left(\alpha \right),\\ \; & =\Delta_{c}{\hat{a}}^{†}\hat{a}+\frac{\Delta_{eg}}{2}{\hat{\sigma }}_{z}+g\left({\hat{\sigma }}_{+}\hat{a}+{\hat{\sigma }}_{-}{\hat{a}}^{†}\right)+\alpha \left(\alpha \Delta_{c}-2\xi \right)\\ \; & \;\;\;+\left(\zeta -g\alpha \right)\left({\hat{\sigma }}_{+}+{\hat{\sigma }}_{-}\right)+\left(\xi -\alpha \Delta_{c}\right)\left(\hat{a}+{\hat{a}}^{†}\right). \end{array}$$

Under the matching conditions α=ζ/g and $\Delta_{c}=\xi /\alpha$, the Hamiltonian maps exactly onto the stationary Jaynes–Cummings form:

(4) $${\hat{H}}_{D}=\Delta_{c}{\hat{a}}^{†}\hat{a}+\frac{\Delta_{eg}}{2}{\hat{\sigma }}_{z}+g\left({\hat{\sigma }}_{+}\hat{a}+{\hat{\sigma }}_{-}{\hat{a}}^{†}\right)-\frac{\zeta \xi }{g}\equiv {\hat{H}}_{\mathrm{JCM}}-\frac{\zeta \xi }{g}.$$

The exact time-evolution operator for the original driven system, ${\hat{U}}_{\mathrm{DJCM}}\left(t\right)$, is obtained by reversing the transformation sequence:

(5) $${\hat{U}}_{\mathrm{DJCM}}\left(t\right)={\hat{T}}^{†}\left(t\right){\hat{D}}^{†}\left(\alpha \right){\hat{U}}_{\mathrm{JCM}}\left(t\right)\hat{D}\left(\alpha \right),\;{\hat{U}}_{\mathrm{JCM}}\left(t\right)=e^{-i{\hat{H}}_{\mathrm{JCM}}t}.$$

The constant term −ζξ/g in Equation (4) introduces only a global phase to the state vector, representing a constant energy shift that does not influence the system’s internal dynamics. The dynamical invariant for the driven system is identified as $\hat{I}\left(t\right)={\hat{T}}^{†}\left(t\right){\hat{D}}^{†}\left(\alpha \right){\hat{N}}_{\mathrm{exc}}\hat{D}\left(\alpha \right)\hat{T}\left(t\right)$, where ${\hat{N}}_{\mathrm{exc}}={\hat{a}}^{†}\hat{a}+{\hat{\sigma }}_{z}/2$ is the conserved total excitation number of the undriven system [24]. The unitary transformations that define this invariant are precisely those used to map the original time-dependent Hamiltonian onto its stationary counterpart. This invariant-based approach has proven effective in describing a wide range of light–matter systems with non-stationary interactions and beam propagation phenomena [26,27].

The factorization in Equation (5) provides a framework for evaluating system observables in the Heisenberg picture. By expressing the time-evolved operators in terms of the standard Jaynes–Cummings evolution, we obtain the following:

(6a) $$\begin{array}{cc} \hat{a}\left(t\right)={\hat{U}}_{\mathrm{DJCM}}^{†}\left(t\right)\hat{a}\left(t\right){\hat{U}}_{\mathrm{DJCM}}\left(t\right) & =e^{-i\omega_{0}t}\left({\hat{D}}^{†}\left(\alpha \right){\hat{a}}_{\mathrm{JCM}}\left(t\right)\hat{D}\left(\alpha \right)-\alpha \right), \end{array}$$

(6b) $$\begin{array}{cc} {\hat{\sigma }}_{-}\left(t\right)={\hat{U}}_{\mathrm{DJCM}}^{†}\left(t\right){\hat{\sigma }}_{-}\left(t\right){\hat{U}}_{\mathrm{DJCM}}\left(t\right) & =e^{-i\omega_{0}t}{\hat{D}}^{†}\left(\alpha \right){\hat{\sigma }}_{-}^{\mathrm{JCM}}\left(t\right)\hat{D}\left(\alpha \right), \end{array}$$

where ${\hat{O}}_{\mathrm{JCM}}\left(t\right)={\hat{U}}_{\mathrm{JCM}}^{†}\left(t\right)\;\hat{O}\;{\hat{U}}_{\mathrm{JCM}}\left(t\right)$ represents the time-evolved operator. This representation offers a computational advantage for calculating the two-time correlation functions required to determine the time-dependent physical spectrum [29].

### The Jaynes–Cummings Hamiltonian in the Block Diagonal Basis

To evaluate the two-time correlation function, it is advantageous to exploit the symmetries and diagonalization of the standard JCM. Since ${\hat{H}}_{\mathrm{JCM}}$ commutes with ${\hat{N}}_{\mathrm{exc}}={\hat{a}}^{†}\hat{a}+{\hat{\sigma }}_{z}/2$, the corresponding Hilbert space decomposes into an infinite set of invariant subspaces $H_{n}$, each spanned by the basis states {|e,n〉,|g,n+1〉}. Within each *n*-th excitation subspace, the Hamiltonian is represented by the 2×2 matrix:

(7) $$\begin{array}{c} H_{\mathrm{JCM}}^{\left(n\right)}=\left(\begin{array}{cc} \Delta_{c}n+\frac{\Delta_{eg}}{2} & g\sqrt{n+1}\\ g\sqrt{n+1} & \Delta_{c}\left(n+1\right)-\frac{\Delta_{eg}}{2} \end{array}\right) \end{array},\;with\;\begin{array}{cc} \Delta_{c} & =\omega_{c}-\omega_{0},\\ \Delta_{eg} & =\omega_{eg}-\omega_{0}, \end{array}$$

where $g\sqrt{n+1}$ is the quantum Rabi frequency characteristic of the *n*-th manifold. Diagonalizing the above matrix yields the eigenenergies, written as follows:

(8) $$\begin{array}{cc} E_{n}^{\left(\pm \right)} & =\Delta_{c}\left(n+\frac{1}{2}\right)\pm \frac{1}{2}\sqrt{{\left(\Delta_{eg}-\Delta_{c}\right)}^{2}+4g^{2}\left(n+1\right)},\\ \; & =\Delta_{c}\left(n+\frac{1}{2}\right)\pm \frac{1}{2}\sqrt{{\left(\omega_{eg}-\omega_{c}\right)}^{2}+4g^{2}\left(n+1\right)}, \end{array}$$

and eigenstates (also known as polaritonic states)

(9a) $$\begin{array}{cc} |+,n\rangle & =\cos\left(\frac{\theta_{n}}{2}\right)|e,n\rangle +\sin\left(\frac{\theta_{n}}{2}\right)|g,n+1\rangle , \end{array}$$

(9b) $$\begin{array}{cc} |-,n\rangle & =\sin\left(\frac{\theta_{n}}{2}\right)|e,n\rangle -\cos\left(\frac{\theta_{n}}{2}\right)|g,n+1\rangle . \end{array}$$

Here, the mixing angle $\theta_{n}$ quantifies the degree of entanglement of light–matter and is determined by the ratio of the coupling strength to the detuning as $\tan\left(\theta_{n}\right)=\frac{2g\sqrt{n+1}}{\omega_{eg}-\omega_{c}}$. Equations (9a) and (9b) establish the representation of the propagator. The states |±,n〉 diagonalize ${\hat{U}}_{\mathrm{JCM}}\left(t\right)=\exp\left(-i{\hat{H}}_{\mathrm{JCM}}t\right)$. The operator consists of the sum of projectors onto the manifolds, weighted by phase factors, written as follows:

(10) $${\hat{U}}_{\mathrm{JCM}}\left(t\right)=e^{i\frac{\Delta_{eg}t}{2}}|g,0\rangle \langle g,0|+\sum_{n=0}^{\infty }\left[e^{-iE_{n}^{\left(+\right)}t}|+,n\rangle \langle +,n|+e^{-iE_{n}^{\left(-\right)}t}|-,n\rangle \langle -,n|\right].$$

Equation (10) provides the solution for the evolution in the manifold basis. Although the drive introduces the displacement $\hat{D}\left(\alpha \right)$ (where $\alpha =\zeta /g=/\xi /\Delta_{c}$) from Equations (6a) and (6b) and renders the propagator non-diagonal, the decomposition remains the foundation for the derivation of the dynamics. This representation enables the mapping of the state vector and the calculation of the correlation function, which constitute the basis for the study of the spectrum.

## 3. The Time-Dependent Physical Spectrum

The time-dependent physical spectrum, defined by Eberly and Wódkiewicz [29], is given by the following equation:

(11) $$S\left(\Gamma ,\omega ;t\right)=2\Gamma e^{-2\Gamma t}\int_{0}^{t}dt_{1}\int_{0}^{t}dt_{2}\;e^{\left(\Gamma -i\omega \right)t_{1}}e^{\left(\Gamma +i\omega \right)t_{2}}\langle \hat{A}\left(t_{1}\right)\hat{B}\left(t_{2}\right)\rangle ,$$

where $\langle \hat{A}\left(t_{1}\right)\hat{B}\left(t_{2}\right)\rangle$ is the two-time correlation function of the system operators for an initial state |ψ(0)〉, and Γ is the band half-width of the filter. The emission dynamics are determined by the spectral response of the atomic and electromagnetic field subsystems. In this work, the system is initialized in |ψ(0)〉=|e〉⊗|β〉≡|e,β〉, i.e., an excited atom and a coherent field state of amplitude β. For different initial atomic states, we refer to [39].

Using the operator transformations in Equations (6a) and (6b), the dynamics is mapped into the standard Jaynes–Cummings frame. The correlation functions are thus expressed in terms of the coherent state |γ〉≡|α+β〉. The field and atomic correlation functions are as follows:

(12) $$\begin{array}{cc} \langle {\hat{a}}^{†}\left(t_{1}\right)\hat{a}\left(t_{2}\right)\rangle & =e^{i\omega_{0}\left(t_{1}-t_{2}\right)}\langle e,\beta |\left({\hat{D}}^{†}\left(\alpha \right){\hat{a}}_{\mathrm{JCM}}^{†}\left(t_{1}\right)\hat{D}\left(\alpha \right)-\alpha \right)\left({\hat{D}}^{†}\left(\alpha \right){\hat{a}}_{\mathrm{JCM}}\left(t_{2}\right)\hat{D}\left(\alpha \right)-\alpha \right)|e,\beta \rangle ,\\ \; & =e^{i\omega_{0}\left(t_{1}-t_{2}\right)}\langle e,\beta |{\hat{D}}^{†}\left(\alpha \right)[{\hat{a}}_{\mathrm{JCM}}^{†}\left(t_{1}\right){\hat{a}}_{\mathrm{JCM}}\left(t_{2}\right)-\alpha \left({\hat{a}}_{\mathrm{JCM}}^{†}\left(t_{1}\right)+{\hat{a}}_{\mathrm{JCM}}\left(t_{2}\right)\right)\\ & \;\;\;\;\;-\alpha^{2}]\hat{D}\left(\alpha \right)|e,\beta \rangle ,\\ \; & =e^{i\omega_{0}\left(t_{1}-t_{2}\right)}\langle e,\gamma |\left[{\hat{a}}_{\mathrm{JCM}}^{†}\left(t_{1}\right){\hat{a}}_{\mathrm{JCM}}\left(t_{2}\right)-\alpha \left({\hat{a}}_{\mathrm{JCM}}^{†}\left(t_{1}\right)+{\hat{a}}_{\mathrm{JCM}}\left(t_{2}\right)\right)+\alpha^{2}\right]|e,\gamma \rangle , \end{array}$$

where $\alpha =\zeta /g=\xi /\Delta_{c}$ links the driven Hamiltonian in (2) to the Jaynes–Cummings form in Equation (4). Similarly, the atomic correlation function is obtained as follows:

(13) $$\begin{array}{cc} \langle {\hat{\sigma }}_{+}\left(t_{1}\right){\hat{\sigma }}_{-}\left(t_{2}\right)\rangle \; & =e^{i\omega_{0}\left(t_{1}-t_{2}\right)}\langle e,\beta |\left({\hat{D}}^{†}\left(\alpha \right){\hat{\sigma }}_{+}^{\mathrm{JCM}}\left(t_{1}\right)\hat{D}\left(\alpha \right)\right)\left({\hat{D}}^{†}\left(\alpha \right){\hat{\sigma }}_{-}^{\mathrm{JCM}}\left(t_{2}\right)\hat{D}\left(\alpha \right)\right)|e,\beta \rangle ,\\ \; & =e^{i\omega_{0}\left(t_{1}-t_{2}\right)}\langle e,\beta |{\hat{D}}^{†}\left(\alpha \right){\hat{\sigma }}_{+}^{\mathrm{JCM}}\left(t_{1}\right){\hat{\sigma }}_{-}^{\mathrm{JCM}}\left(t_{2}\right)\hat{D}\left(\alpha \right)|e,\beta \rangle ,\\ \; & =e^{i\omega_{0}\left(t_{1}-t_{2}\right)}\langle e,\gamma |{\hat{\sigma }}_{+}^{\mathrm{JCM}}\left(t_{1}\right){\hat{\sigma }}_{-}^{\mathrm{JCM}}\left(t_{2}\right)|e,\gamma \rangle . \end{array}$$

These expressions utilize the displacement operator property $\hat{D}\left(\alpha \right)|\beta \rangle =\hat{D}\left(\alpha \right)\hat{D}\left(\beta \right)|0\rangle =|\alpha +\beta \rangle$ with real amplitudes α,β∈R. This mapping determines the correlation functions for the effective state |γ〉=|α+β〉, where β is the initial field amplitude, and $\alpha =\zeta /g=\xi /\Delta_{c}$ links the driven Hamiltonian in (2) to the Jaynes–Cummings form in (4). We will show that by tuning the displacement to cancel the initial field amplitude (α=−β) we recover the vacuum Rabi splitting in the emission spectrum.

Although the preceding formalism applies to both the field and atomic subsystems, we focus our analysis on the atomic spectral response to clearly highlight the light–matter interaction signatures. Accordingly, the final analytical expression for the time-dependent atomic physical spectrum is (see Appendix A for a detailed derivation):

(14) $$S_{\mathrm{atom}}\left(\Gamma ,\omega ;t\right)=2\Gamma e^{-2\Gamma t}\left[S_{\mathrm{vac}}\left(t\right)+\sum_{n=1}^{\infty }S_{n}\left(t\right)\right].$$

The vacuum contribution (n=0) results from decay in the ground state. Laboratory frequencies $\nu_{0}^{\left(\pm \right)}=\omega_{0}+E_{0}^{\left(\pm \right)}+\Delta_{eg}/2$ lead to the following:

(15) $$S_{\mathrm{vac}}\left(t\right)=e^{-{\left|\gamma \right|}^{2}}{\left|\cos^{2}\left(\frac{\theta_{0}}{2}\right)L\left(\nu_{0}^{\left(+\right)}-\omega ;t\right)+\sin^{2}\left(\frac{\theta_{0}}{2}\right)L\left(\nu_{0}^{\left(-\right)}-\omega ;t\right)\right|}^{2},$$

where

(16) $$L\left(\delta ;t\right)\equiv \frac{e^{(\Gamma +i\delta )t}-1}{\Gamma +i\delta }.$$

The contribution of excited manifolds (n≥1) results from the four transition paths with $\nu_{n}^{\left(j,k\right)}=\omega_{0}+E_{n}^{\left(j\right)}-E_{n-1}^{\left(k\right)}$:

(17) $$\begin{array}{cc} S_{n}\left(t\right) & =P_{n}[\sin^{2}\left(\frac{\theta_{n-1}}{2}\right){\left|\cos^{2}\left(\frac{\theta_{n}}{2}\right)L\left(\nu_{n}^{\left(+,+\right)}-\omega ;t\right)+\sin^{2}\left(\frac{\theta_{n}}{2}\right)L\left(\nu_{n}^{\left(-,+\right)}-\omega ;t\right)\right|}^{2}\\ \; & +\cos^{2}\left(\frac{\theta_{n-1}}{2}\right){\left|\cos^{2}\left(\frac{\theta_{n}}{2}\right)L\left(\nu_{n}^{\left(+,-\right)}-\omega ;t\right)+\sin^{2}\left(\frac{\theta_{n}}{2}\right)L\left(\nu_{n}^{\left(-,-\right)}-\omega ;t\right)\right|}^{2}], \end{array}$$

where $P_{n}=e^{-{\left|\gamma \right|}^{2}}{\left|\gamma \right|}^{2n}/n!$, and

(18) $$\begin{array}{cc} \sin^{2}\left(\frac{\theta_{n}}{2}\right) & =\frac{1}{2}\left(1-\frac{\omega_{eg}-\omega_{c}}{\sqrt{{\left(\omega_{eg}-\omega_{c}\right)}^{2}+4g^{2}\left(n+1\right)}}\right),\\ \cos^{2}\left(\frac{\theta_{n}}{2}\right) & =\frac{1}{2}\left(1+\frac{\omega_{eg}-\omega_{c}}{\sqrt{{\left(\omega_{eg}-\omega_{c}\right)}^{2}+4g^{2}\left(n+1\right)}}\right). \end{array}$$

**Figure 2:**
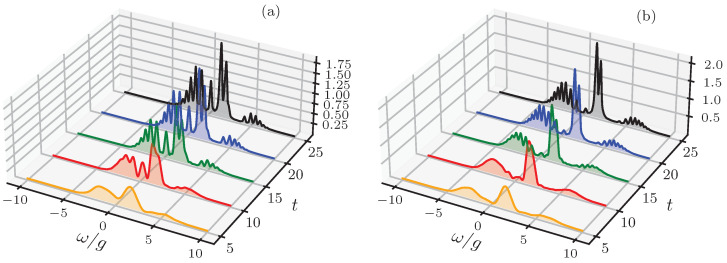
Temporal evolution of the atomic physical spectrum $S_{\mathrm{atom}}\left(\Gamma ,\omega ;t\right)$ in Equation (14), highlighting the progressive emergence of dressed-state resonances across multiple interaction timescales. At incipient times, the spectral response is characterized by a broad, unresolved profile, a direct consequence of the Fourier-limited resolution intrinsic to the Eberly–Wódkiewicz windowing. As the interaction time increases ($t\gg \Gamma^{-1}$), the transient frequency fluctuations are exponentially suppressed, allowing the spectrum to resolve into a discrete set of sharp resonances associated with transitions between polaritonic manifolds in the Jaynes–Cummings ladder. The observable peak asymmetries and the underlying oscillatory modulations reflect the interplay between the off-resonant coupling and the coherent displacement induced by the external drive. Simulation parameters: g=1.0, $\omega_{0}=1.95$, $\omega_{eg}=1.00$, $\omega_{c}=1.75$, β=3, and Γ=0.1. Panel (**a**) corresponds to a weak-driving scenario with ξ=0.3 ($\alpha =\zeta /g=\xi /\Delta_{c}\approx -1.5$), while panel (**b**) illustrates the substantial spectral reshaping occurring in the high-driving regime with ξ=1.0 (α≈−5.0).

**Figure 3:**
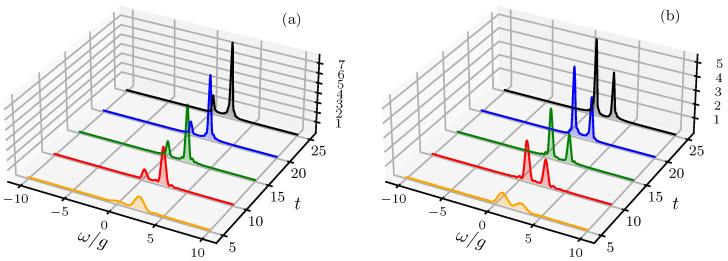
Atomic physical spectrum, $S_{\mathrm{atom}}\left(\Gamma ,\omega ;t\right)$ in Equation (14), as a function of ω and *t*, demonstrating the recovery of the vacuum Rabi splitting. The results illustrate that the external driving mechanism facilitates the exact coherent cancellation of the initial cavity field when the effective coherence parameter vanishes (γ=0 for β=−α). Case (**a**) corresponds to a cavity frequency $\omega_{c}=1.0$ (leading to an atomic drive amplitude $\alpha =\zeta /g=\xi /\Delta_{c}\approx -1.05$), while case (**b**) examines the regime with $\omega_{c}=2.0$ ($\alpha =\zeta /g=\xi /\Delta_{c}\approx 19.0$). In both scenarios, the suppression of higher-order Mollow-like sidebands reveals the underlying vacuum Rabi doublet, highlighting a controllable transition from a driven many-photon regime to a vacuum-mediated light–matter interaction. Simulation parameters: g=1.0, $\omega_{0}=1.95$, $\omega_{eg}=1.75$, ξ=1.0, and Γ=0.1.

The analytical results derived in Equation (14) provide a comprehensive framework for characterizing the multi-manifold emission dynamics of the driven JCM, which we elucidate through the atomic physical spectra displayed in Figure 2 and Figure 3. In Figure 2, we illustrate the progressive emergence of spectral resolution as a function of interaction time *t*; at early stages (Γt≈1, the spectrum is dominated by broad overlapping profiles due to the Fourier-limited resolution of the Eberly–Wódkiewicz windowing, whereas in the asymptotic regime (Γt≫1), transient frequency fluctuations are exponentially suppressed, revealing discrete resonances associated with transitions between polaritonic manifolds. A salient feature in this temporal evolution is the pronounced asymmetry in the spectral peak heights, which constitutes a fundamental signature of off-resonant coupling. For finite detuning ($\omega_{eg}\neq \omega_{c}$), the dressed states {|±,n〉} acquire unequal atomic and photonic components—as governed by the mixing angles $\theta_{n}$ in Equation (9a)—thereby favoring transition paths with higher atomic weighting. This imbalance reflects the structural properties of the light–matter entanglement, where the emission process preferentially tracks the atomic population distribution across the Jaynes–Cummings ladder.

Figure 3 shows the recovery of the vacuum limit through the cancellation of the field amplitude. When the drive satisfies the condition β=−α, with $\alpha =\zeta /g=\xi /\Delta_{c}$, the effective coherence parameter vanishes (γ=0). This condition results from the destructive interference between the external drive and the initial cavity field, formulated through the unitary displacement D(α) in the rotating frame. This cancellation maps the driven two-time correlation function in Equation (13) to the undriven vacuum case and restricts the system evolution to the Jaynes–Cummings dynamics in the displaced basis. Within this framework, the time-dependent dynamical invariant recovers its undriven algebraic structure, formulated explicitly as follows:

(19) $$\hat{I}\left(t\right)={\hat{T}}^{†}\left(t\right){\hat{D}}^{†}\left(\alpha \right)\left({\hat{a}}^{†}\hat{a}+\frac{{\hat{\sigma }}_{z}}{2}\right)\hat{D}\left(\alpha \right)\hat{T}\left(t\right),$$

while the effective Hamiltonian becomes time-independent in the rotating frame. The Poissonian weights $P_{n}$ in $S_{n}\left(t\right)$ of Equation (14) collapse to the Kronecker delta $\delta_{n,0}$, which reduces the system response from a weighted sum over the infinite ladder of dressed-state manifolds to a single-excitation subspace. The weights of all manifolds n≥1, which scale as ${\left|\gamma \right|}^{2n}$ according to the Poisson distribution, are zero, and the interaction is confined to transitions between the polaritonic doublet {|+,0〉,|−,0〉} and the ground state |g,0〉 in the displaced frame. The spectral response is defined by these transitions, which produce a doublet at frequencies $\omega =\omega_{0}\pm \sqrt{\Delta^{2}/4+g^{2}}+\Delta_{eg}/2$ (with $\Delta =\omega_{eg}-\omega_{c}$). For the resonant case ($\omega_{eg}=\omega_{c}$), the peak separation remains fixed at the Rabi vacuum frequency $\Omega_{0}\equiv 2g$. The intensities of these resonances are determined by the mixing angle $\theta_{0}$, where the detuning Δ introduces a height asymmetry corresponding to the weighting of the atomic population of the dressed states. Specifically, the spectral intensities are defined by the analytical weights $W_{0}^{\left(\pm \right)}=\frac{1}{4}{\left(1\pm \Delta /\Omega_{0}\right)}^{2}$ (with $\Delta =\omega_{eg}-\omega_{c}$), which dictate the emission rate in the zero-photon sector from the upper and lower polaritons, respectively. These weights arise from the projection of the atomic transition operator onto the polaritonic eigenbasis of the dynamical invariant $\hat{I}\left(t\right)$ and reflect the degree of mixing of light–matter. In this limit, the multi-manifold interference that generates the sidebands in the γ≠0 regime is suppressed because the field enters the dynamics through the vacuum state fluctuations. This mechanism suppresses the Mollow-like structures and isolates the vacuum-field coupling signatures at the single-photon level. As the interaction time *t* increases in this regime, the detector bandwidth Γ resolves the underlying energy levels of the Jaynes–Cummings ladder, leading to a reduction in the Lorentzian linewidths. The displacement acts as a control parameter to cancel out the mean field amplitude and resolve the vacuum-mediated splitting in an externally driven system where the driving effect is nullified in the rotating frame defined by the invariant framework of transformations. This situation allows for the investigation of fundamental vacuum effects in the presence of strong external fields.

### Steady-State Physical Spectrum

The transient dynamics shown in Figure 2 resolve into a stationary structure in the long-time limit Γt≫1. To characterize the steady-state emission, we evaluate the limit of Equation (14) as t→∞. Using the definition of the windowing function L(δ;t) in Equation (14) (see Equation (16)), the relevant term for the spectral intensity scales is as follows:

(20) $$2\Gamma e^{-2\Gamma t}{\left|L\left(\delta ;t\right)\right|}^{2}=\frac{2\Gamma \left(1-2e^{-\Gamma t}\cos\left(\delta t\right)+e^{-2\Gamma t}\right)}{\Gamma^{2}+\delta^{2}}.$$

In the limit t→∞ the exponential terms vanish and the normalized windowing function converges to a Lorentzian profile, written as follows:

(21) $$\lim_{t\to \infty }2\Gamma e^{-2\Gamma t}{\left|L\left(\delta ;t\right)\right|}^{2}=\frac{2\Gamma }{\Gamma^{2}+\delta^{2}},$$

with a full-width at half-maximum determined by the photodetector bandwidth 2Γ. Although the time-dependent spectrum in Equations (15) and (17) involves the absolute square of a sum of windowing functions, the stationary representation relies on the resolved peak approximation. When the separation between polaritonic resonances Δν satisfies Δν≫Γ, the spectral overlap between distinct Lorentzian profiles is negligible. Under these conditions, the cross-interference terms in $|\sum_{j}c_{j}L_{j}{|}^{2}$ vanish, and the steady-state atomic physical spectrum reduces to a weighted sum of independent Lorentzian peaks:

(22) $$\begin{array}{cc} S_{\mathrm{atom}}^{\infty }\left(\Gamma ,\omega \right) & =2\Gamma e^{-{\left|\gamma \right|}^{2}}\left[W_{0}^{\left(+\right)}\frac{1}{\Gamma^{2}+{\left(\nu_{0}^{\left(+\right)}-\omega \right)}^{2}}+W_{0}^{\left(-\right)}\frac{1}{\Gamma^{2}+{\left(\nu_{0}^{\left(-\right)}-\omega \right)}^{2}}\right]\\ \; & +2\Gamma \sum_{n=1}^{\infty }P_{n}\sum_{j,k\in \{+,-\}}W_{n}^{\left(j,k\right)}\frac{1}{\Gamma^{2}+{\left(\nu_{n}^{\left(j,k\right)}-\omega \right)}^{2}}, \end{array}$$

where the spectral weights $W_{0}^{\left(\pm \right)}$ and $W_{n}^{\left(j,k\right)}$ absorb the squared amplitudes

(23) $$\begin{array}{cc} W_{0}^{\left(\pm \right)} & =\frac{1}{4}{\left(1\pm \frac{\Delta }{\Omega_{0}}\right)}^{2},\\ W_{n}^{\left(j,\pm \right)} & =\frac{1}{8}\left(1\mp \frac{\Delta }{\Omega_{n-1}}\right){\left(1\pm \frac{\Delta \delta_{j,+}-\Delta \delta_{j,-}}{\Omega_{n}}\right)}^{2}, \end{array}$$

with $\Delta =\omega_{eg}-\omega_{c}$ and $\Omega_{n}=\sqrt{\Delta^{2}+4g^{2}\left(n+1\right)}$. The weights $P_{n}$ represent the Poissonian distribution of the excitation manifolds. This stationary representation highlights that the multi-peak structure observed in Figure 2 remains robust in the steady state, where the detector resolution Γ acts as the effective linewidth of the polaritonic resonances. In the vacuum limit (β=−α), the sum in Equation (22) collapses to the vacuum Rabi doublet, where the intensities reflect the degree of light–matter mixing at the single-excitation level.

## 4. Conclusions

In this work, we presented an analytical characterization of the time-dependent physical spectrum for the driven Jaynes–Cummings model. Our approach, grounded in the invariant framework [24], allowed for an exact algebraic treatment of the driven dynamics by mapping the time-dependent Hamiltonian onto a stationary block-diagonal form. This methodology provided closed-form expressions for the atomic and field correlation functions, allowing the evaluation of the Eberly–Wódkiewicz spectrum [29] across all time scales.

A fundamental insight gained from this analysis is the interpretation of the external drive as a coherent field displacement $\hat{D}\left(\alpha \right)$, which reshapes the photon-number distribution and uniquely modifies the spectral weights of the dressed-state ladder. We demonstrated that for the driving conditions (β=−α), the mean-field fluctuations are coherently nullified, effectively mapping the system dynamics to the undriven vacuum limit. This mechanism provides a transparent tool for isolating vacuum-field signatures in the presence of strong external fields, where the emission spectrum simplifies to the vacuum Rabi splitting.

In the asymptotic long-time limit (Γt≫1), we derived the steady-state atomic spectrum as a weighted sum of Lorentzian peaks. By invoking the resolved peak approximation—where the polaritonic separation exceeds the detector bandwidth—we established a justification for neglecting cross-interference terms between distinct resonances. The resulting representation highlights that the stationary asymmetry in peak heights is a structural property of the light–matter entanglement, determined by algebraic weights $W_{n}^{\left(j,k\right)}$ that quantify the weighting of the atomic population within each excitation manifold.

Beyond its theoretical significance, the formulation presented here provides a versatile framework for exploring time-resolved spectral control in modern cavity QED and circuit QED architectures [39], where coherent driving and strong light–matter coupling are routinely achieved [3,4]. The connection identified between coherent-field cancellation and the suppression of multi-manifold interference points to potential applications in quantum state engineering and recently proposed concepts such as quantum catalysis, in which light–matter interactions mediate the controlled redistribution of quantum fluctuations [40]. The present approach also admits natural extensions to driven–dissipative scenarios and to the inclusion of squeezed or other non-classical initial field states, which are expected to further broaden the applicability of the invariant-based spectral analysis developed here. From an experimental perspective, the predicted spectral tunability lies well within the capabilities of current quantum platforms: in circuit QED, superconducting qubits coupled to microwave resonators enable precise and independent control of driving amplitudes, detunings, and coherence times, while high-resolution transmission and emission spectroscopy has already revealed dressed-state spectra and drive-induced modifications of light–matter coupling [3,4]; similarly, trapped-ion systems provide an alternative realization of driven Jaynes–Cummings-type dynamics, where internal electronic states coupled to motional modes can be coherently driven and spectrally probed with high precision, allowing the effective displacement induced by the external drive to be accurately tuned and making the observation of the predicted spectral reshaping experimentally feasible with existing technology.

## Data Availability

No new data were created or analyzed in this study.

## Appendix A. Derivation of the Time-Dependent Physical Spectrum

In this appendix, we outline the exact derivation of the time-dependent atomic physical spectrum. The starting point is the two-time correlation function in the Heisenberg picture $\langle {\hat{\sigma }}_{+}\left(t_{1}\right){\hat{\sigma }}_{-}\left(t_{2}\right)\rangle =e^{i\omega_{0}\left(t_{1}-t_{2}\right)}\langle \Phi \left(t_{1}\right)|\Phi \left(t_{2}\right)\rangle$, where we define the atomic source state vector as follows:

(A1) $$|\Phi (t)\rangle ={\hat{U}}_{\mathrm{JCM}}^{†}\left(t\right){\hat{\sigma }}_{-}{\hat{U}}_{\mathrm{JCM}}\left(t\right)|e,\gamma \rangle .$$

To exactly evaluate this vector, we expand the initial effective state $|e,\gamma \rangle =\sum_{n=0}^{\infty }C_{n}|e,n\rangle$ (with $C_{n}=e^{-{\left|\gamma \right|}^{2}/2}\gamma^{n}/\sqrt{n!}$) in terms of the dressed eigenstates {|+,n〉,|−,n〉} of each manifold *n*. Using the mapping rules ${\hat{\sigma }}_{-}|+,n\rangle =c_{n}|g,n\rangle$ and ${\hat{\sigma }}_{-}|-,n\rangle =s_{n}|g,n\rangle$, where $c_{n}=\cos\left(\theta_{n}/2\right)$ and $s_{n}=\sin\left(\theta_{n}/2\right)$, the forward and backward time evolution produces the following explicit form:

(A2) $$|\Phi (t)\rangle =C_{0}f_{0}\left(t\right)e^{i\frac{\Delta_{eg}}{2}t}|g,0\rangle +\sum_{n=1}^{\infty }C_{n}f_{n}\left(t\right)\left[s_{n-1}e^{iE_{n-1}^{\left(+\right)}t}|+,n-1\rangle -c_{n-1}e^{iE_{n-1}^{\left(-\right)}t}|-,n-1\rangle \right],$$

with the dynamical amplitudes defined as $f_{n}\left(t\right)=c_{n}^{2}e^{-iE_{n}^{\left(+\right)}t}+s_{n}^{2}e^{-iE_{n}^{\left(-\right)}t}$. The orthogonality of the dressed-state manifolds ensures that the inner product $\langle \Phi \left(t_{1}\right)|\Phi \left(t_{2}\right)\rangle$ collapses into a sum over the independent manifolds $P_{n}={\left|C_{n}\right|}^{2}$. By substituting the laboratory transition frequencies $\nu_{n}^{\left(j,k\right)}=\omega_{0}+E_{n}^{\left(j\right)}-E_{n-1}^{\left(k\right)}$, the correlation function becomes a superposition of oscillating terms, written as follows:

(A3) $$G_{\mathrm{atom}}\left(t_{1},t_{2}\right)=P_{0}\sum_{j\in \{+,-\}}W_{0}^{\left(j\right)}e^{i\nu_{0}^{\left(j\right)}\left(t_{1}-t_{2}\right)}+\sum_{n=1}^{\infty }P_{n}\sum_{j,k\in \{+,-\}}W_{n}^{\left(j,k\right)}e^{i\nu_{n}^{\left(j,k\right)}\left(t_{1}-t_{2}\right)}.$$

The physical spectrum S(Γ,ω;t) is then obtained by inserting $G_{\mathrm{atom}}$ into the Eberly–Wódkiewicz integral in Equation (11). Because each frequency component $\nu_{n}^{\left(j,k\right)}$ factorizes the double time integral into the product $|L\left(\omega -\nu_{n}^{\left(j,k\right)};t\right){|}^{2}$, the final spectrum resolves into the weighted sum provided in Equation (14).

The spectral weights $W_{n}^{\left(j,k\right)}$ quantify the probabilities of atomic transition between the dressed-state ladders. These weights emerge from the squared amplitudes of the source state vector in Equation (A2). For the vacuum contribution (n=0), the transition occurs from the excited state |e,0〉 to the absolute ground state |g,0〉, mediated by the two polariton branches |±,0〉. The corresponding weights are as follows:

(A4) $$W_{0}^{\left(\pm \right)}={\left|c_{0}^{2}\pm s_{0}^{2}\right|}^{2}=\frac{1}{4}{\left(1\pm \frac{\Delta }{\Omega_{0}}\right)}^{2},$$

where $\Omega_{0}=\sqrt{\Delta^{2}+4g^{2}}$ is the vacuum Rabi frequency and $\Delta =\omega_{eg}-\omega_{c}$ is the atom-cavity detuning. These weights describe the asymmetry of the vacuum Rabi doublet: at resonance (Δ=0), both peaks have equal intensity ($W_{0}^{\left(+\right)}=W_{0}^{\left(-\right)}=1/4$), while off-resonance, one branch dominates over the other.

For excited manifolds (n≥1), the atomic lowering operator induces transitions from manifold *n* to manifold n−1. Each transition involves four possible pathways, corresponding to the initial and final polariton indices (j,k)∈{+,−}×{+,−}. The weights are as follows:

(A5) $$W_{n}^{\left(j,k\right)}=\frac{1}{8}\left(1-k\frac{\Delta }{\Omega_{n-1}}\right){\left(1+j\frac{\Delta }{\Omega_{n}}\right)}^{2},$$

where $\Omega_{n}=\sqrt{\Delta^{2}+4g^{2}\left(n+1\right)}$ is the generalized Rabi frequency for the *n*-th manifold. The first factor, $(1-k\Delta /\Omega_{n-1})/2$, represents the atomic character of the initial polariton state in the manifold n−1, while the second factor, ${\left(1+j\Delta /\Omega_{n}\right)}^{2}/4$, encodes the squared transition amplitude of the manifold *n*. This factorization reflects the selection rules of the atomic dipole operator in the dressed basis.

The detuning-dependent structure of these weights is responsible for the spectral asymmetries observed in Figure 2 and Figure 3. Specifically, when Δ>0 (atom black-detuned from the cavity), the upper polariton branch (j=+) acquires a larger atomic component, enhancing the emission probability for transitions involving this state. In contrast, for Δ<0, the lower branch (j=−) dominates. This asymmetry is a fundamental signature of light–matter hybridization and is directly encoded in the algebraic form of $W_{n}^{\left(j,k\right)}$.
